# Oral health-related quality of life in children and adolescent with autism spectrum disorders and neurotypical peers: a nested case–control questionnaire survey

**DOI:** 10.1007/s40368-024-00970-y

**Published:** 2024-11-08

**Authors:** C. Salerno, G. Campus, G. Bontà, G. Vilbi, G. Conti, M. G. Cagetti

**Affiliations:** 1 Department of Biomedical, Surgical and Dental Sciences, University of Milan, Via Beldiletto 1, 20142 Milan, Italy; 2 Department of Restorative, Preventive and Pediatric Dentistry, University of Bern, Freiburgstrasse 7, 3012 Bern, Switzerland; 3 Graduate School for Health Sciences, University of Bern, Bern, Switzerland; 4 Department of Medicine and Surgery, University of Insubria, 21100 Varese, Italy; 5 Department of Cariology, Institute of Odontology, Sahlgrenska Academin University of Gothenburg, Gothenburg, Sweden

**Keywords:** Oral-Health-Related-Quality-Of-Life, Autism, Children, Parents

## Abstract

**Purpose:**

Oral health-related quality of life (OHRQoL) of children with autism following an oral preventive regimen (ASDsP) was compared to that of children with autism without a preventive regimen (ASDsNP) and of neurotypical peers (NT).

**Methods:**

An online survey was carried out using the Parental/Caregiver Perception Questionnaire (P-CPQ) and the Family Impact Scale (FIS). Scores were assigned to items, with the total score ranging from 0 to 120 (worst QoL). The median scores were compared amongst groups using the Mann–Whitney and Kruskal–Wallis tests. A multivariate linear regression assessed the relationship between questionnaire scores and demographical variables.

**Results:**

Overall, 168 questionnaires from the ASDsP and the ASDsNP groups, respectively, and 336 from the NT group were selected ASDsP compared to ASDsNP showed lower P-CPQ Emotional wellbeing, FIS Emotion (*p* < 0.01) and Conflict (*p* < 0.05), and FIS total score (*p* < 0.01). In addition, they were less nervous (*p* < 0.05), shy (*p* < 0.01), with better sleep (*p* < 0.05), and with happier parents (*p* < 0.01). Compared to NT, ASDsP showed higher P-CPQ + FIS, P-CPQ, and FIS total scores (*p* < 0.01) and P-CPQ Functional limitation, Social wellbeing (*p* < 0.01), Emotional wellbeing (*p* < 0.05), and FIS Activity, Emotion and Conflict (*p* < 0.01).

**Conclusion:**

The preventive regimen reduces parental stress, improving the quality of life of children and families.

**Supplementary Information:**

The online version contains supplementary material available at 10.1007/s40368-024-00970-y.

## Introduction

Oral health involves the ability to speak, smile, taste, feel, chew, swallow, and convey emotions confidently through facial expressions without pain, discomfort, and illness of the craniofacial complex (Constitution of the World Health Organization, 2011). Quality of life (QoL) is defined as the individual’s perception of one’s position in life in the context of the culture and value systems in which one lives and about goals, expectations, standards, and worries. QoL is a valuable indicator in evaluating physical and mental health, including oral health (The World Health Organization Quality of Life assessment (WHOQOL): position paper from the World Health Organization, 1995). Oral health-related quality of life (OHRQoL) affects overall health and wellbeing. The World Health Organization recognised OHRQoL as an essential element of the Global Oral Health Program (Petersen 2003). The OHRQoL encompasses several aspects, such as chewing, sleep, school performance, social interactions, and self-esteem. These can be summarised into four main domains: Oral symptoms, Psychological wellbeing, Activity, and Social wellbeing (Bennadi and Reddy 2013). Several questionnaires measuring the OHRQoL have been proposed and validated in different languages and used in public health policy, research, and clinical practice (Page et al., 2019). Under the age of six, the Early Childhood Oral Health Impact Scale (ECOHIS) is the most widely used (Thomson et al., 2014), while the Child Oral Health Related Quality of Life measures OHRQoL in older children. The latter includes two questionnaires: the Parental-Caregivers Perception Questionnaire (P-CPQ), which deals with the impact of oral health on the child, and the Family Impact Scale (FIS), which deals with the effects of a patient’s oral health on the family.

Dental caries can affect an individual’s oral health and, consequently, overall health (Filstrup et al. 2003), causing pain, discomfort, acute and chronic infections, and, in the case of children, loss of school days, affecting learning and schooling. These problems are common in the general population but even more prevalent in adults and children with special needs, in whom poor health can contribute to worsening QoL (G. F. Ferrazzano et al. 2020; Pani et al. 2013).

Autism Spectrum Disorders (ASDs) are complex biological disorders that generally last a lifetime and are characterised by social and communication impairments, restricted interests, and repetitive behaviour (American Psychiatric Association 2013). Like other children with chronic illnesses, children with ASDs experience an impaired quality of life (Sheldrick et al. 2012). Although autism does not have a direct effect on oral health, it is often associated with severe difficulties in performing oral hygiene procedures at home and preventive/therapeutic procedures at the dental chair, exposing affected individuals to an increased risk of dental caries, gingivitis, periodontal disease, and traumatic injuries (Bagattoni et al. 2021; G. F. Ferrazzano et al. 2020). In addition, parents of children with ASDs reported more difficulties with their children’s access to dental care than parents of peers without ASDs (Du et al. 2015). All these problems contribute to worsening the oral health status of children with autism, potentially affecting their quality of life (Nqcobo et al., 2019). However, the studies investigating OHRQoL in children with ASDs are scarce, with a limited number of participants (Pani et al. 2013; da Silva et al., 2023). Autism, as a spectrum disorder, can manifest itself with significant heterogeneity of manifestations, and the lack of studies with an adequate sample size could potentially mask the specific oral health challenges faced by subgroups within the affected population. Moreover, no study has investigated whether an oral prevention programme can improve their OHRQoL. Recently, the dental caries experience was shown to impact OHRQoL in a group of children/adolescents with ASDs regarding the total P-CPQ score and the Oral symptoms and Wellbeing domains if compared to a sample of neurotypical peers. However, no differences were reported between the perceptions of oral health by parents/caregivers (Procopio et al. 2023). Few studies have reported a lower OHRQoL perceived by parents of children and adolescents with ASDs when compared to those of neurotypical peers (Pani et al. 2013; da Silva et al., 2023). The P-CPQ scores are generally worse than those of neurotypical peers, except in the Oral symptoms domain, where no significant difference was observed (Pani et al. 2013; da Silva et al., 2023). This lack of difference can be attributed to the objectivity of the Oral symptoms domain, which assesses concrete indicators like pain, gingival bleeding, and halitosis, which are less susceptible to parental influence. Conversely, Emotional and Social wellbeing seem to be influenced by the behavioural aspects associated with ASDs, affecting how parents of these children respond. Significantly higher Family Impact Scale (FIS) scores were also reported by parents/caregivers of children with ASDs, indicating a considerable impact on family routines. This impact extends to disrupted sleep for parents and potential work absences, underscoring the broader consequences of the child’s or adolescent’s oral health condition on the family dynamic ^14,15^(Filstrup et al. 2003, García et al. 2020)

It has been shown that specifically structured oral health preventive programmes for children with ASDs performed by qualified dentists have led to better cooperation during dental visits and treatments, improved oral hygiene, and a low incidence of caries lesions (Balian et al., 2022). However, to date, to the best of the authors’ knowledge, no study has investigated the effect of a preventive programme dedicated to special needs children and adolescents, such as those with ASDs, on OHRQoL.

Starting from these premises, an online survey was designed, in which the OHRQoL of children and adolescents with ASDs following an oral health preventive regimen (ASDsP) was compared to the OHRQoL of children and adolescents with ASDs with no oral health regimen (ASDsNP) and to the OHRQoL of neurotypical peers (NT).

## Methods

The study was designed as a comparative cross-sectional study with protocol approval by the Ethics Committee Board of San Paolo Hospital, Milan, Italy (number 1041/2022) and conducted following the Declaration of Helsinki. The study population consisted of three groups: children and adolescents with ASDs receiving an oral health preventive regimen at the Dental Clinic of San Paolo Hospital, Milan, Italy, as ASDsP group, compared to children and adolescents with ASDs but not enrolled in an oral health preventive regimen, as ASDsNP group, and to a neurotypical peers group, as NT group. According to the Dental Clinic protocol for children with disabilities, as they are considered at high risk of caries, parents receive oral hygiene instructions and dietary recommendations during the admission visit and follow-up visits, and children receive regular oral hygiene sessions, application of 5% sodium fluoride varnish quarterly, fissures sealants on permanent molars and caries treatment if needed. A toothpaste with at least 1000 ppm of fluoride is also recommended. Two post-graduate students in Paediatric Dentistry with at least 2 years of training in children with special needs perform all dental procedures.

The inclusion criteria for the ASDsP group were parents/caregiver with a child aged between 6 and 18 years with ASDs, with no other comorbidities, enrolled amongst patients included in the preventive programme; for ASDsNP group were: parents/caregiver with a child aged between 6 and 18 years with ASDs, with no others comorbidities, who were not patient in the Dental Clinic of San Paolo Hospital and were not enrolled in any preventive programme; finally, for NT group were: parents/caregiver with a child aged between 6 and 18 years without ASDs and with no others comorbidities and not enrolled in any preventive programme. All parents/caregivers had to have signed the consent form before accessing the questionnaire.

For sample size calculation, the possibility of non-responders was considered. Due to the absence of data on the quality of life in subjects with autism inserted in a standardised oral prevention programme, the OHRQoL scores of children with autism and unaffected children were taken into account to calculate the ES (Pani et al. 2013). With an ES = 0.33, a significance of 95%, and a power of 90%, the calculation resulted in 121 children and adolescents for the ASDsP and ASDsNP groups and 241 for the NT group.

From the total number of compiled questionnaires, once the age of children matched the sample, an appropriate number of questionnaires was randomly selected to reach the enrolment ratio. Randomisation was performed on an Excel spreadsheet (Microsoft Excel 2019 for Mac), using the random command, sorting the subjects in ascending order, and selecting the appropriate number of subjects starting from the first in the list. Only questionnaires from parents/caregivers of children aged between 6 and 18 were considered. Data were collected between April and September 2022.

### Questionnaires

The short-form version of the Parental/Caregiver Perception Questionnaire (P-CPQ) and the Family Impact Scale Questionnaire (FIS) were used (Thomson et al. 2013).

The P-CPQ, consisting of 16 questions, evaluates children’s oral health and its effect on their overall wellbeing; it is divided into four domains: Oral symptoms, Functional limitations, Emotional wellbeing, and Social wellbeing. Scores were assigned to each item using a five-point Likert scale, classifying the responses into numerical values as don’t know = 0, never = 1, once or twice = 2, sometimes = 3, often = 4, every day or almost every day = 5. The total P-CPQ questionnaire score is the sum of all scores obtained and ranges from 0 to 80. The higher the score is, the poorer the quality of life is.

The FIS questionnaire consists of 8 items on the impact of oral health on the family’s quality of life. It is divided into three domains: Family activities, Parental emotions, and Family conflicts. The total score of the FIS ranges from 0 to 40, and the answer options with their corresponding scores are the same as those reported above for the P-CPQ; the higher the score, the poorer the quality of life.

A few questions were added to the standardised questionnaires to collect responders’ demographical and socioeconomic data and the parent’s evaluation of the child’s oral health and its impact on the child’s overall health (see Supplementary file S1). The parents’ assessment of the child’s oral health was categorised with the following scores: 1 = poor; 2 = insufficient; 3 = sufficient; 4 = above sufficient; 5 = excellent), while the impact on the overall health was scored as: 1 = not at all; 2 = a little; 3 = sufficiently; 4 = enough; 5 = very.

As no validated Italian versions of the questionnaires were available, the standardised questionnaires were translated from English into Italian by two native Italian-speaking translators fluent in English and experienced in the topic. After the translation, a consensus version was identified and then retranslated by a native English-speaking person who was not involved in the study to ensure the accuracy and comparability of the translation. Furthermore, a comparison was made for the corresponding items with the Italian version of the CPQ11-14 (Olivieri et al. 2013). The translated questionnaires were pre-tested for comprehensibility on a small sample of 20 parents/caregivers not included in the study sample. After completion, the subjects were contacted to find out whether they had experienced any difficulty in understanding the questions, assigning a comprehension score from 1 (extreme difficulty) to 5 (no difficulty) with a mean score ± standard error of 4.40 ± 0.34.

The final questionnaire included additional items to collect data on children’s age and demographical and socioeconomic characteristics of the parents as the person who filled the questionnaire, age, educational level, employment status, type of house, family income and source of family income, to investigate confounding variables that could affect the quality of life. The online questionnaire was prepared using Google Form (Google LLC, Mountain View, CA, USA) and shared by e-mail with parents/caregivers of children with ASDs following an oral health preventive regimen at the Dental Clinic of San Paolo Hospital, Milan, Italy. An ad hoc flyer containing the QR code of the questionnaire was created to enrol children with and without ASDs who are not receiving the preventive regimen at the Dental Clinic. The flyer was made available in the waiting rooms of several family doctors, paediatricians, and dentists. All potential parents and/or legal guardians received a description of the purpose of the study and were asked to sign an online informed consent, following Italian data protection laws. All potential participants were asked some questions before being submitted to the questionnaire to check for meeting the inclusion and exclusion criteria. Subjects who did not fit into the groups considered could not access the questionnaire. The questionnaire was automatically closed if they did not sign the consent form.

### Statistical analysis

All data obtained from the included questionnaires were entered into a spreadsheet (Excel™ 2019 for Mac) for statistical processing and analysis. The Shapiro–Wilk test and Levene’s test assessed the normality and heteroscedasticity of data. The median of the scores for each question, based on a Likert scale, were analysed and compared between two groups (children with ASDs regardless of the inclusion in a preventive oral regimen (ASDsT) and neurotypical peers (NT)) using Mann–Whitney and between the three groups (the two groups of children with ASDs vs. the neurotypical group) using Kruskal–Wallis test. If the null hypothesis of Kruskal–Wallis’ test was rejected, *post-hoc* pairwise analysis was performed using Dunn–Bonferroni’s test. For the trend analysis, the scores were categorised based on quartiles as follows: P-CPQ Oral symptoms, P-CPQ Functional limitation, P-CPQ Emotional wellbeing, P-CPQ Social wellbeing, FIS Parental emotion (1–5 = Low; 6–10 = Medium; > 10 = High); FIS Family activity and FIS Family conflict (1–3 = Low; 4–6 = Medium; > 6 = High). Cuzick’s test was performed to investigate differences in the trend of scores for each domain of the two questionnaires. A multivariable linear regression was performed to assess the relation between the P-CPQ and FIS scores and the explanatory variables: parent’s age, educational level, employment status, and family income. Data were checked for multicollinearity with the Belsley–Kuh–Welsch technique (García et al. 2020). The heteroscedasticity and normality of residuals were assessed using the White and the Shapiro–Wilk tests. Alpha was set to 5%.

## Results

Overall, 909 includable questionnaires were collected: 168 from parents/caregivers of children and adolescents with characteristics ascribable to the ASDsP group, 174 to the ASDsNP group, and 567 to the NT group. Starting from 168 questionnaires collected for the ASDsP group, a randomisation procedure was performed with an enrolment ratio of 1:1:2. The subjects were nested according to age, and the enrolment ratio was set to compare subjects with and without ASDs. So, 168 questionnaires were randomly selected from the ASDsNP group and 336 from the NT group.

Demographic and socioeconomic characteristics of the parents are presented considering parents of children with ASDs *versus* parents of neurotypical peers. The sex of the respondents was predominantly female in both groups. However, the sex of the children differed significantly between the two groups due to a higher male representation in the groups of children with ASDs (*p* < 0.01). Children with autism had older parents than neurotypical peers (mean age 46.06 *vs* 43.00; *p* < 0.01), although children’s age was paired (Table 1).

**Table 1:** Descriptive characteristics on demographical and socioeconomic data amongst ASDsT [ASDsP = children with ASDs inserted in a preventive regimen (n = 168) and ASDsNP = children with ASDs not inserted in a preventive regimen (n = 168)] and NT = neurotypical children (n = 336)

	ASDs (Total)	NT	
	*n* = 336	*n* = 336	*p*-value
	Items related to family/parents		
Parent’s age Mean ± SD [range]	46.06 ± 7.36 [27–73]	43.00 ± 5.42[27–56]	< 0.01^a^
Who filled in the questionnaire n (%)			
Mother	265 (78.87)	320 (95.24)	< 0.01^b^
Father	68 (20.24)	16 (4.76)	
Legal guardian	3 (0.89)	0 (0.00)	
Educational level n (%)			
Primary school diploma	34 (10.12)	17 (5.06)	0.09^b^
Middle school diploma	157 (46.73)	176 (52.38)	
High-school diploma	19 (5.65)	13 (3.87)	
University degree	121 (36.01)	126 (37.50)	
Post-graduate qualifications	5 (1.49)	4 (1.19)	
Employment status n (%)			
Worker	230 (68.45)	261 (77.68)	< 0.01^b^
Irregular worker	14 (4.17)	25 (7.44)	
Unemployed	92 (27.38)	50 (14.88)	
Type of house n (%)			
Property house	273 (81.25)	288 (85.71)	0.29^c^
Rented house	60 (17.86)	45 (13.39)	
Public housing	3 (0.89)	3 (0.90)	
Family income n (%)			
Less than € 15.000	66 (19.64)	33 (9.82)	< 0.01^b^
Between € 15.001 and € 25.000	86 (25.60)	90 (26.79)	
Between € 25.001 and € 40.000	101 (30.06)	125 (37.20)	
Between € 40.001 and € 60.000	39 (11.60)	62 (18.45)	
Major than € 60.000	44 (13.10)	26 (7.74)	
Source of family income n (%)			
Salary	279 (83.04)	333 (99.10)	< 0.01^c^
Accompanying allowance	46 (13.69)	2 (0.60)	
Retiring allowance	11 (3.27)	1 (0.30)	
	Item related to child		
Child’s age Mean ± SD [Range]	11.02 ± 4.20 [6–18]	10.63 ± 2.73 [6–18]	0.92^a^

Differences amongst groups were evaluated with

^a^ = Mann–Whitney test

^b^ = Chi squared test

^c^ = Fisher’s exact test

Forty-three questionnaires containing more than three “I don’t know” answers were excluded from the analysis. Of these, 25 (14.88%) belonged to the ASDsP, 11 (6.55%) to the ASDsNP group, and 7 (2.08%) to the NT group. Nearly one-third (31.55%) of parents of children with autism were unemployed or had irregular employment compared to 22.32% of parents of neurotypical peers. Significant differences between groups were found in the distribution of family income (*p* < 0.01). Regarding the source of income, families of children with ASDs received higher benefits from the Italian State than those of neurotypical children (16.96% *vs* 0.90%; *p* < 0.01).

Data on the kind of dentist and check-up frequency were only analysed for the ASDsNP and NT, excluding the ASDsP group due to the inclusion criteria (data not in table). Regarding neurotypical children, 81.85% visited a private dentist, 7.44% visited a public dentist, and 10.71% did not visit the dentist at all. In the ASDsNP group, 42.86% of children visited a private dentist, 25.60% a public dentist, and 31.55% did not visit the dentist at all. The comparison between the groups showed significant differences (*p* < 0.01). Demographic and socioeconomic characteristics amongst the three groups are presented in Supplementary file S2.

Concerning the parents’ perception of the child’s oral health and its impact on general health, the worst scores were obtained by the ASDsP group (Table 2).

**Table 2:** Parents’ perception on child’s oral health status in the three groups: *ASDsP* = children with ASDs inserted in a preventive regimen (*n* = 168), ASDsNP = children with ASDs not inserted in a preventive regimen (*n* = 168), *NT* = neurotypical children (*n* = 336) and *ASDsT* = ASDsP = children with ASDs inserted in a preventive regimen (*n* = 168) and *ASDsNP* = children with ASDs not inserted in a preventive regimen (*n* = 168)

Variable	Group			
	ASDsP	ASDsNP	NT	ASDsT
How would you classify the health of your child’s teeth, lips, jaw and mouth				
Mean ± SD^§#$*^^	2.88 ± 0.77	3.11 ± 0.91	3.38 ± 0.80	2.99 ± 0.85
(1 = poor; 2 = insufficient; 3 = sufficient; 4 = above sufficient; 5 = excellent)				
How much was your child’s general wellbeing influenced by the state of his teeth, lips, jaw or mouth				
Mean ± SD^§$^^	2.61 ± 1.07	2.48 ± 1.22	2.24 ± 1.10	2.54 ± 1.15
(1 = not at all; 2 = a little; 3 = sufficiently; 4 = enough; 5 = very)				

Differences between 2 groups were evaluated with Mann–Whitney test, between 3 groups with Kruskal–Wallis’s test

§ statistically significant between ASDsP vs ASDsNP and vs NT

# statistically significant between ASDsP vs ASDsNP

$ statistically significant between ASDsP vs NT

* statistically significant between ASDsNP vs NT

^ statistically significant between ASDsT vs NT

Table 3 shows the average scores for each item, for each domain, and the total scores of the P-CPQ and FIS. Statistically significant differences were found amongst groups in items included in the domains of the P-CPQ Functional limitation, Emotional wellbeing, Social wellbeing, and domains of the FIS. Halitosis was reported more frequently in ASDsNP, with a significant difference between the two groups with autism (*p* = 0.03). Children with ASDs, compared to NT, reported greater difficulty in chewing solid foods and sleeping. In the area of emotional wellbeing, about the emotions of nervousness/fear/anxiety (2.79 ± 1.03 *vs* 3.13 ± 1.10; *p* = 0.04) and shyness/embarrassment (1.89 ± 1.04 *vs* 2.28 ± 1.18; *p* < 0.01), the ASDsP group was judged to be less afflicted than children in the ASDsNP In the area of social wellbeing, no differences were found between the two groups with ASDs. Although the NT group scored lower than the two groups for both the total FIS score and the different FIS domains, the ASDsP group scored lower in all the above-mentioned aspects when compared to the ASDsNP group. In particular, the largest differences were found in both the total FIS (20.59 ± 5.06 *vs* 22.31 ± 5.57; *p* < 0.01) and the FIS Emotion domain (11.40 ± 3.36 *vs* 12.50 ± 3.41; *p* < 0.01). The P-CPQ Emotional wellbeing was also statistically lower in the ASDsP than in the ASDsNP group (11.40 ± 3.36 *vs* 12.50 ± 3.41; *p* < 0.01).

**Table 3:** Items distribution in the three groups: ASDsP children with ASDs inserted in a preventive regimen (*n* = 168), ASDsNP children with ASDs not inserted in a preventive regimen (*n* = 168), NT neurotypical children (*n* = 336) and ASDsT total children with ASDs

	P-CPQ				
	Items	ASDsP	ASDsNP	NT	ASDsT
		*(n) Mean* ± *SD*	*(n) Mean* ± *SD*	*(n) Mean* ± *SD*	*(n) Mean* ± *SD*
Oral symptoms	Halitosis	(138) 2.06 ± 1.09	(154) 2.23 ± 1.19	(321) 2.02 ± 1.08	(292) 2.15 ± 1.15
	Tooth, mouth, lip or jaw pain^§$*^^	(137) 1.86 ± 1.04	(143) 1.88 ± 1.02	(328) 1.62 ± 0.85	(280) 1.87 ± 1.03
	Food stuck to the palate^§$^^	(137) 1.31 ± 0.76	(150) 1.23 ± 0.67	(327) 1.10 ± 0.41	(287) 1.27 ± 0.72
	Food stuck amongst teeth	(139) 2.22 ± 1.11	(147) 2.14 ± 1.13	(323) 2.14 ± 0.94	(286) 2.18 ± 1.12
	*Oral symptoms total*	*(143) 7.17* ± *2.74*	*(157) 7.09* ± *2.65*	*(329) 6.79* ± *2.14*	*(300) 7.13* ± *2.69*
Functional limitation	Difficulty chewing solid food^§$*^^	(142) 1.55 ± 0.97	(155) 1.61 ± 1.08	(328) 1.27 ± 0.67	(297) 1.58 ± 1.03
	Mouth breathing	(140) 1.98 ± 1.26	(143) 2.04 ± 1.23	(315) 1.80 ± 1.05	(283) 2.01 ± 1.24
	Eating slower / Taking longer than others	(142) 1.82 ± 1.25	(157) 1.94 ± 1.33	(325) 1.84 ± 1.24	(299) 1.89 ± 1.29
	Trouble sleeping^§$*^^	(143) 2.39 ± 1.33	(157) 2.39 ± 1.28	(329) 1.54 ± 0.88	(300) 2.39 ± 1.30
	*Functional limitation total*^§$*^^	*(143) 7.68* ± *3.07*	*(157) 7.79* ± *3.24*	*(329) 6.35* ± *2.45*	*(300) 7.74* ± *3.15*
Emotional wellbeing	Irritable or frustrated^§$*^^	(142) 2.92 ± 0.98	(156) 3.10 ± 1.01	(327) 2.25 ± 1.01	(298) 3.01 ± 1.00
	Sad^§#*^^	(139) 2.20 ± 0.95	(154) 2.51 ± 1.03	(329) 2.15 ± 0.89	(293) 2.37 ± 1.00
	Nervous, fearful or anxious^§#$*^^	(143) 2.79 ± 1.03	(157) 3.13 ± 1.10	(329) 2.33 ± 0.97	(300) 2.97 ± 1.08
	Timid/embarrassed^§#$^^	(141) 1.89 ± 1.04	(155) 2.28 ± 1.18	(329) 2.33 ± 1.04	(296) 2.09 ± 1.13
	*Emotional wellbeing total*^§#$*^^	*(143) 9.69* ± *2.66*	*(157) 10.93* ± *3.35*	*(329) 9.04* ± *2.89*	*(300) 10.34* ± *3.10*
Social wellbeing	He/She skipped school^§$*^^	(142) 1.84 ± 1.10	(157) 1.75 ± 0.90	(329) 1.49 ± 0.76	(299) 1.79 ± 1.00
	He/She avoided smiling in front of other children^§$*^^	(138) 1.64 ± 1.06	(146) 1.57 ± 1.0	(328) 1.20 ± 0.58	(284) 1.61 ± 1.04
	He/She had difficulty concentrating in class^§$*^^	(135) 3.26 ± 1.13	(140) 3.42 ± 1.11	(315) 1.97 ± 1.08	(275) 3.34 ± 1.12
	He/She didn’t want to talk to other children^§$*^^	(125) 2.64 ± 1.32	(137) 2.63 ± 0.34	(326) 1.28 ± 0.61	(262) 2.63 ± 1.33
	*Social wellbeing total*^§$*^^	*(143) 8.80* ± *3.06*	*(157) 8.55* ± *3.16*	*(329) 5.84* ± *1.98*	*(300) 8.67* ± *3.11*
	P-CPQ^§$*^^	*(143) 33.34* ± *7.99*	*(157) 34.36* ± *8.97*	*(329) 28.01* ± *6.69*	*(300) 33.88* ± *8.52*
	**FIS**				
Activity	Time off work^§$*^^	(142) 2.87 ± 1.18	(157) 2.80 ± 1.13	(329) 1.74 ± 0.87	(299) 2.83 ± 1.15
	Request of more attention^§$*^^	(142) 3.11 ± 1.28	(155) 3.35 ± 1.29	(327) 1.81 ± 0.96	297) 3.23 ± 1.29
	* Activity total*^§$*^^	(143) 5.93 ± 2.00	(157) 6.10 ± 1.98	(329) 3.53 ± 1.45	(300) 6.02 ± 1.99
Emotion	Less time for yourselves or for others^§$*^^	(143) 3.39 ± 1.21	(157) 3.64 ± 1.23	(328) 2.35 ± 1.16	(300) 3.52 ± 1.23
	Disturbed sleep^§#$*^^	(142) 2.85 ± 1.17	(157) 3.15 ± 1.22	(328) 2.24 ± 1.11	(299) 3.01 ± 1.21
	Feeling sad^§#$*^^	(143) 2.91 ± 0.94	(157) 3.20 ± 0.99	(326) 2.40 ± 0.93	(300) 3.06 ± 0.98
	Feeling guilty^§*^^	(141) 2.30 ± 1.14	(156) 2.54 ± 1.19	(326) 2.16 ± 1.05	(297) 2.42 ± 1.17
	* Emotion total*^§#$*^^	(143) 11.40 ± 3.36	(157) 12.50 ± 3.41	(329) 9.09 ± 3.29	(300) 11.98 ± 3.43
Conflict	Arguing with the child^§$^	(137) 2.14 ± 1.11	(156) 2.38 ± 1.16	(327) 2.43 ± 0.98	(293) 2.27 ± 1.14
	Blamed by your child^§$*^^	(133) 1.30 ± 0.80	(148) 1.43 ± 0.89	(325) 1.66 ± 0.91	(281) 1.37 ± 0.85
	* Conflict total*^§#$*^^	*(143) 3.26* ± *1.72*	*(157) 3.71* ± *1.75*	*(329) 4.05* ± *1.66*	*(300) 3.49* ± *1.75*
	FIS^§#$*^^	*(143) 20.59* ± *5.06*	*(157) 22.31* ± *5.57*	*(329) 16.68* ± *5.19*	*(300) 21.49* ± *5.39*
	P-CPQ + FIS^§$*^^	*(143) 53.93* ± *11.22*	*(157) 56.68* ± *12.99*	*(329) 44.69* ± *10.53*	*(300) 55.37* ± *12.24*

Differences between 2 groups were evaluated with Mann–Whitney test, between 3 groups with Kruskal–Wallis’s test

§ statistically significant between ASDsP vs ASDsNP and vs NT

# statistically significant between ASDsP vs ASDsNP

$statistically significant between ASDsP vs NT

* statistically significant between ASDsNP vs NT

^ statistically significant between ASDsT vs NT. If no symbol is reported after each item or domain, the p value was greater than 0.05 for each comparison performed

A linear trend was observed amongst the three groups, starting from the NT to the ASDsP and the ASDsNP. Cuzick’s test results showed a trend of the scores for P-CPQ Functional limitation (*z* = 5.73), Emotional wellbeing (*z* = 4.64), Social wellbeing (*z* = 9.16), and FIS Activity (*z* = 13.28) and Emotion (*z* = 8.47). In contrast, an inverse trend regarding the FIS Conflict domain (*z* = −2.17) was observed (Table 4). A higher proportion of subjects with lower scores was found in the NT compared to the ASDsP and the ASDsNP. The ASDsNP showed a higher proportion of subjects in the highest score rank, indicating a worse quality of life than the ASDsP and the NT.

**Table 4:** Sub-areas questionnaire score trend within groups

	NT	ASDsP	ASDsNP
	*n (%)*	*n (%)*	*n (%)*
P-CPQ Oral symptoms			
Low (1–5)	101 (30.70)	39 (20.97)	46 (24.37)
Moderate (6–10)	216 (65.65)	81 (56.64)	93 (59.24)
High (> 10)	12 (3.65)	23 (16.08)	18 (11.46)
*Cuzick’s trend* = *9.11* ± *5.20 z* = *1.76 p* = *0.08*			
P-CPQ Functional limitation			
Low (1–5)	141 (42.86)	38 (26.57)	34 (21.66)
Moderate (6–10)	166 (50.46)	78 (54.55)	93 (59.24)
High (> 10)	22 (6.69)	27 (18.88)	30 (19.11)
*Cuzick’s trend* = 31.10 ± *5.43 z* = *5.73 p* < *0.01*			
P-CPQ Emotional wellbeing			
Low (1–5)	47 (14.29)	10 (6.99)	10 (6.37)
Moderate (6–10)	173 (52.58)	78 (54.55)	61 (39.49)
High (> 10)	109 (33.13)	55 (38.46)	85 (54.14)
*Cuzick’s trend* = 25.34 ± *5.63 z* = *4.64 p* < *0.01*			
P-CPQ Social wellbeing			
Low (1–5)	165 (50.15)	24 (16.78)	30 (19.11)
Moderate (6–10)	155 (47.11)	78 (54.55)	87 (55.41)
High (> 10)	9 (2.74)	41 (28.67)	40 (25.48)
*Cuzick’s trend* = 50.30 ± *5.49 z* = *9.16 p* < *0.01*			
FIS Activity			
Low (1–3)	174 (52.89)	18 (12.59)	17 (10.83)
Moderate (4–6)	150 (45.59)	68 (47.55)	66 (42.04)
High (> 7)	5 (1.52)	57 (39.86)	74 (47.13)
*Cuzick’s trend* = 74.58 ± *5.62 z* = *13.28 p* < *0.01*			
FIS Emotion			
Low (1–5)	52 (15.81)	8 (5.59)	4 (2.55)
Moderate (6–10)	160 (48.63)	45 (31.47)	38 (24.20)
High (> 10)	117 (35.56)	90 (62.94)	115 (73.25)
*Cuzick’s trend* = 46.09 ± *5.44 z* = *8.47 p* < *0.01*			
FIS Conflict			
Low (1–3)	124 (37.92)	81 (58.27)	73 (46.50)
Moderate (4–6)	181 (55.35)	48 (34.53)	73 (46.50)
High (> 7)	22 (6.73)	10 (7.19)	11 (7.01)
*Cuzick’s trend* = −1.37 ± *5.39 z* = *−2.17 p* = *0.03*			

*ASDsP* = children with ASDs inserted in a preventive regimen (*n* = 168), *ASDsNP* = children with ASDs not inserted in a preventive regimen (*n* = 168), *NT* = Neurotypical children (*n* = 336)

The effect of different sociodemographic factors on parents’ perceptions of their children’s quality of life was analysed using a logistic regression model with the P-CPQ as the dependent variable (Table 5). The degree of association between the different sociodemographic variables was demonstrated by the constant (*p* < 0.01 for each group) (Table 5). The coefficients were used to show the degree and direction of association (positive values indicated a direct association, while negative correlations indicated an inverse association). Family income had a significant influence in the opposite direction on the P-CPQ score in the ASDsP and the NT. In this last group, parents’ age had a significant negative impact on P-CPQ score (*p* < 0.01); higher values of P-CPQ were associated with older parents. The FIS was used as the dependent variable in the same model to study the overall effects of sociodemographic factors on the family, but no statistical association was found.

**Table 5:** Multiple logistic regression

Model	Group	Variable	Coefficient [CI]	*p*-Value
P-CPQ	ASDsP	Constant	36.18 [26.48; 45.88]	< 0.01
		Parent’s age		
		Risk for each 1-unit increase	−0.09 [−0.28; 0.09]	0.31
		Educational level		
		Ref: Diploma or inferior		
		University or superior	1.98 [−0.77; 4.73]	0.16
		Employment status		
		Ref: Worker		
		Unemployed	−2.82 [−5.65; 0.01]	0.05
		Family income		
		Ref: Between € 25.001 and € 40.000		
		Less than € 15.000	5.44 [1.06; 9.82]	0.01
		Between € 15.001 and € 25.000	4.01 [0.73; 7.29]	0.02
		Between € 40.001 and € 60.000	−5.00 [-8.47; −1.53]	< 0.01
		Major than € 60.000	0.40 [−5.06; 5.84]	0.88
	ASDsNP	Constant	25.82 [16.27; 35.37]	< 0.01
		Parent’s age		
		Risk for each 1-unit increase	0.18 [−0.02; 0.38]	0.07
		Educational level		
		Ref: Diploma or inferior		
		University or superior	−1.59 [−4.58; 1.41]	0.30
		Employment status		
		Ref: Worker		
		Unemployed	−0.48 [−3.66; 2.71]	0.77
		Family income		
		Ref: Between € 25.001 and € 40.000		
		Less than € 15.000	4.23 [−0.35; 8.81]	0.07
		Between € 15.001 and € 25.000	2.18 [−1.69; 6.04]	0.27
		Between € 40.001 and € 60.000	0.32 [−4.50; 5.13]	0.90
		Major than € 60.000	−2.02 [−6.76; 2.71]	0.40
	NT	Constant	22.59 [17.53; 27.65]	< 0.01
		Parent’s age		
		Risk for each 1-unit increase	0.16 [0.05; 0.27]	< 0.01
		Educational level		
		Ref: Diploma or inferior		
		University or superior	−0.27 [−1.60; 1.07]	0.69
		Employment status		
		Ref: Worker		
		Unemployed	0.76 [−0.70; 2.23]	0.30
		Family income		
		Ref: Between € 25.001 and € 40.000		
		Less than € 15.000	4.84 [2.56; 7.11]	< 0.01
		Between € 15.001 and € 25.000	2.56 [0.94; 4.18]	< 0.01
		Between € 40.001 and € 60.000	−0.81 [−2.56; 0.94]	0.36
		Major than € 60.000	−1.10 [−3.66; 1.45]	0.40

The P-CPQ was used as dependent variables to analyse the effects of sociodemographic variables on parental perceptions and family impact

## Discussion

Oral hygiene procedures are often referred to parents/caregivers of children with ASDs, and difficulties in performing these procedures can facilitate the development of caries and gingivitis (Bagattoni et al., 2021). In children, including those with special needs, socio-demographical characteristics of the family play an important role in oral health (Bombert et al., 2018). In the present survey, parents of children and adolescents with ASDs judged worse OHRQoL compared to neurotypical peers; however, following a prevention regimen seems to reduce parents’ stress, improving both family and children’s quality of life.

Women were the primary respondents to the study questionnaire, as reported in similar studies on the OHRQoL (Abanto et al. 2014) and self-assessment questionnaires in general (Curtin et al. 2000). However, in the sample of parents of children with autism, a quarter of the respondents were male, whereas in the group of parents of neurotypical peers, the male share was much less represented. Older parental age is a well-known risk factor for autism spectrum disorders; the parents of the sample with autism were significantly older than those of the neurotypical peers, as expected (Lyall et al. 2020).

Parents’ self-assessment of their children’s oral health and the impact of oral health on the child’s general health was assessed with two extra items. Surprisingly, the parents of the children in the ASDsP group gave the worst answers to both questions, probably because this sample consists of parents of patients who had sought dental care before being included in the preventive programme. Moreover, these parents were trained and informed by qualified personnel about their child’s oral health status and the impact of oral health plays on general health, thus contributing to greater awareness in this area. All these elements may have contributed to a harsher judgement in this group.

Over the past decade, health-related quality of life measures and OHRQoL have been highlighted as validated measures for children and adolescents (Gianmaria F. Ferrazzano et al. 2020). However, there are very few studies comparing OHRQoL of children with ASDs to children without ASDs. Parental perceptions and the effects of different sociodemographic variables on the OHRQoL of children with ASDs were previously investigated using the P-CPQ and FIS questionnaires (Pani et al. 2013; Procopio et al. 2023). The studies followed a cross-sectional comparative design in which data of children with ASDs with those of neurotypical children were cross-matched. The results showed that children with ASDs had poorer OHRQoL than neurotypical children. These results are consistent with the present findings: the questionnaire scores of both groups of children with autism were significantly worse than those of the neurotypical peers. However, the OHRQoL of the neurotypical children in this investigation resulted worse than that of the unaffected sibling evaluated by Pani et al., in contrast, that of children with autism here considered was better than that of the siblings with autism (Pani et al. 2013). Having one child with autism and one without may have led parents to overestimate the OHRQoL of the unaffected child and underestimate that of the child with autism. This aspect could be explained by the halo effect to which siblings of individuals with ASDs are often condemned, as parents are focussed too much on the needs of the child with autism, not addressing the needs of the neurotypical child (Garrido et al. 2020).

Functional limitation, Emotional wellbeing, and Social wellbeing scores in the P-CPQ questionnaire were significantly higher in both groups of children with ASDs than in neurotypical peers. At the same time, no differences were found in the Oral symptoms domain. These findings are consistent with those of the literature (Pani et al. 2013), according to which parents of children with autism were more likely to make more severe judgments in the domains affected by disability than in the Oral symptoms domain. The only domain of the P-CPQ that differed between the two groups of children with autism included in the present study was Emotional wellbeing; this could be explained by the effect of the preventive programme that appears to alleviate children’s stress, consequently improving family and children’s quality of life.

Scores of FIS domains were also worse amongst families with children and adolescents with autism than those with neurotypical peers, as found in the literature (Pani et al. 2013). Significant differences were found between the two groups with autism in the FIS Emotions and Conflict domains, which were lower in the group of children with ASDs included in the preventive oral regimen. The risk of burn-out that the presence of a child with autism can cause in parents is well known (Kütük et al. 2021). Knowing that the child is included in a preventive regimen could reduce parents’ stress and improve their quality of life. The family is relieved of the burden of seeking and scheduling regular check-ups with dental personnel specialised in the care of children with ASDs, confirming once again the positive impact that the preventive regimen can have, not only on the child’s oral health, but also on the entire family’s quality of life.

Parents’ age has been reported to play a statistically significant role in P-CPQ and educational level on FIS (Pani et al. 2013), yet in the present survey, parents’ age was statistically significantly associated with P-CPQ scores only in neurotypical children and adolescents. Family income was the sociodemographic variable significantly influencing parental perception (P-CPQ) in ASDsP and NT groups. The studies on neurotypical subjects reported better OHRQoL in children from high-income families and high parental education (Kumar et al. 2014). High income and high education are protective factors for caries (Campus et al., 2022). Therefore, children with more schooled and high-income parents may have fewer caries, better oral health, and a better quality of life, explaining the association in the analysed sample. In families with children with special needs, economic disparities may be less perceived as a limitation due to the more significant burden the disability itself has on quality of life. In the present survey, the prevention regimen may have played a key role in reducing the halo effect of the disability, bringing scores of OHRQoL closer to those of the neurotypical group. These results align with the trends of the scores obtained in the three groups. For almost all domains of both questionnaires, the scores of the group of children with autism followed with a prevention regimen were higher than the scores obtained by the group of neurotypical children and lower than the group of children with autism not included in a preventive regimen. This result further supports the positive impact that a regular oral prevention regimen can have on the quality of life of children and adolescents with ASDs and their families.

One of the strengths of this survey is the large sample, which provides an overview of the impact of oral health on the quality of life of children and adolescents with autism compared to an unaffected population. It is also the first survey that investigates the impact of an oral prevention regimen on the quality of life of individuals with special needs, such as children with ASDs.

### Limitations

The study’s results must be interpreted with caution due to certain limitations, particularly those due to possible sampling and response bias. The sample was recruited using two different methods. Children with ASDs attending an oral prevention programme were enrolled in a single dental clinic and can be considered a convenience sample; however, the study aimed to assess the effectiveness of such a programme on quality of life justified this choice. About the other two groups (neurotypical children and children with ASDs who were not in a prevention programme), enrolment was performed among parents who attended the waiting rooms of different clinicians. Only parents attending the offices where the leaflet was distributed could participate and only parents interested in the problem participated in the survey. This aspect limits the possibility of considering the sample representative of the entire population. However, efforts were made to reach a high number of parents, thanks to a large circulation of the flyer. Furthermore, this sampling method might have produced a response bias as parents who participated may have had more experience with their child’s dental care.

A further limitation is that the children’s oral conditions were not recorded, so it was impossible to associate objective clinical data with the questionnaire results. However, collecting clinical data in such a large sample would have required much time and unavailable funds.

Another aspect to consider is that no validated Italian versions of the questionnaires were available. However, the questionnaires were obtained using a translation and reverse translation method, and many items were the same as the validated Italian version of CPQ11-14 (Olivieri et al. 2013). In addition, the OHRQoL questionnaires were not developed for children with special needs; this may explain the high number of ‘don’t know’ answers given by parents of children with autism to questions concerning the domains of functional limitations and social interactions, as the specific characteristics of the autistic spectrum strongly influence these areas.

## Conclusion

In conclusion, this study sheds light on the significant challenges faced by parents of children with ASDs in managing their oral health, highlighting a worse perceived oral health-related quality of life (OHRQoL) compared to that of neurotypical peers. The results show the positive impact that a regular regimen of preventive oral care can have, not only in improving the oral health-related quality of life of children with ASDs but also in reducing parental stress and improving overall family quality of life.

## Supplementary Information

Below is the link to the electronic supplementary material.

Supplementary file1 (DOCX 15 KB)

Supplementary file2 (DOCX 117 KB)

## Data Availability

The datasets generated and/or analysed during this study can may be released by the corresponding author upon reasonable request.
