# Supplementary material for: Oral health-related quality of life in children and adolescent with autism spectrum disorders and neurotypical peers: a nested case–control questionnaire survey
Source: Eur Arch Paediatr Dent. 2024 Nov 8;26(2):299–310. doi: 10.1007/s40368-024-00970-y (PMC11972231; doi:10.1007/s40368-024-00970-y)
Supplement: Supplementary file 1 — Supplementary file1 (DOCX 15 KB) [file 40368_2024_970_MOESM1_ESM.docx]

SUPPLEMENTARY FILE I:

PARENT-CAREGIVER DEMOGRAPHIC AND SOCIO-ECONOMIC ITEMS

1. What is your Age?

Years

1. What is your sex?

☐Male ☐Female

1. What is your relationship to the child?

☐ Mother ☐Father ☐Legal guardian ☐Other (specify)__________________

1. What is the disability of the child?

☐Down’s syndrome ☐Cerebral Palsy ☐Autism ☐Epilepsy ☐Other (specify) __________________

1. What is your level of education?

☐Primary school ☐Middle school ☐High School ☐College/ University ☐Post-graduate,

1. Where do you and your family stay?

☐Own a house ☐ Rented house ☐Public housing ☐Other, specify____________

1. Employment status

☐ Worker ☐ Irregular worker ☐ Unemployed

1. What is your source of Household income?

☐ Salary ☐Accompanying allowance ☐ Retiring allowance

1. What is the scale of the combined income in your household?

☐ Less than € 15.000 ☐ Between € 15.001 and € 25.000☐ Between € 40.001 and € 60.000☐ Major than € 60.000

1. What is your Child’s Age?

Years

ADDITIONAL ITEMS ON PARENT’S SELF-EVALUATION

1. How would you classify the health of your child's teeth, lips, jaw and mouth?

1=poor; 2=insufficient; 3=sufficient; 4=above sufficient; 5=excellent

1. How much was your child's general well-being influenced by the state of his teeth, lips, jaw or mouth?

1=not at all; 2=a little; 3=sufficiently; 4=enough; 5=very
