# Supplementary material for: Oral health-related quality of life in children and adolescent with autism spectrum disorders and neurotypical peers: a nested case–control questionnaire survey
Source: Eur Arch Paediatr Dent. 2024 Nov 8;26(2):299–310. doi: 10.1007/s40368-024-00970-y (PMC11972231; doi:10.1007/s40368-024-00970-y)
Supplement: Supplementary file 2 — Supplementary file2 (DOCX 117 KB) [file 40368_2024_970_MOESM2_ESM.docx]

**Supplementary file S2**

Descriptive characteristics on demographic and socioeconomic data among ASDs(Total) [ASDsP= children with ASDs inserted in a preventive regimen (n=168) and ASDsNP= children with ASDs not inserted in a preventive regimen (n=168)] and NT=neurotypical children (n=336).

|  | | **ASDsP** | | **ASDsNP** | **NT** |  |
| --- | --- | --- | --- | --- | --- | --- |
|  | | *n = 168* | | *n* = 168 | *n* = 336 | p-value |
|  |  | | **Items related to family/parents** | | |  |
| **Parent’s age** Mean±SD [range] | | 45.70±8.71 [27-70] | | 46.02±7.22 [28-73] | 43.00±5.42 [27-56] | <0.05^a^ |
| **Who filled in the questionnaire** n (%) | |  | |  |  |  |
| Mother | | 130 (77.38) | | 135 (80.36) | 320 (95.24) | <0.05^b^ |
| Father | | 35 (20.83) | | 33 (19.64) | 16 (4.76) |  |
| Legal guardian | | 3 (1.79) | | 0 (0.0) | 0 (0.00) |  |
| **Educational level** n (%) | |  | |  |  |  |
| Primary school diploma | | 17 (10.12) | | 17 (10.12) | 17 (5.06) | 0.08^b^ |
| Middle school diploma | | 78 (46.43) | | 79 (47.02) | 176 (52.38) |  |
| High-school diploma | | 14 (8.33) | | 5 (2.98) | 13 (3.87) |  |
| University degree | | 56 (33.33) | | 65 (38.69) | 126 (37.50) |  |
| Post-graduate qualifications | | 3 (1.79) | | 2 (1.19) | 4 (1.19) |  |
| **Employment status** n (%) | |  | |  |  |  |
| Worker | | 116 (69.05) | | 114 (67.86) | 261 (77.68) | <0.05^b^ |
| Irregular worker | | 10 85.95) | | 4 (2.38) | 25 (7.44) |  |
| Unemployed | | 42 (25.0) | | 50 (29.76) | 50 (14.88) |  |
| **Type of house** n (%) | |  | |  |  |  |
| Property house | | 135 (80.36) | | 138 (82.14) | 288 (85.71) | 0.51^c^ |
| Rented house | | 31 (18.45) | | 29 (17.26) | 45 (13.39) |  |
| Public housing | | 2 (1.19) | | 1 (0.60) | 3 (0.90) |  |
| **Family income** n (%) | |  | |  |  |  |
| Less than € 15.000 | | 38 (22.62) | | 28 (16.67) | 33 (9.82) | <0.05^b^ |
| Between € 15.001 and € 25.000 | | 41 (24.40) | | 45 (26.79) | 90 (26.79) |  |
| Between € 25.001 and € 40.000 | | 50 (29.76) | | 51(30.36) | 125 (37.20) |  |
| Between € 40.001 and € 60.000 | | 18 (10.71) | | 21 (12.50) | 62 (18.45) |  |
| Major than € 60.000 | | 21 (12.50) | | 23 (13.69) | 26 (7.74) |  |
| **Source of family income** n (%) | |  | |  |  |  |
| Salary | | 138 (82.14) | | 141 (83.93) | 333 (99.10) | <0.05^c^ |
| Accompanying allowance | | 23 (13.69) | | 23 (13.69) | 2 (0.60) |  |
| Retiring allowance | | 7 (4.17) | | 4 (2.38) | 1 (0.30) |  |
|  |  | | **Item related to child** | | |  |
| **Child’s age** Mean ±SD (Range) | | 11.39±4.28 [6-18] | | 10.65±4.09 [6-18] | 10.63±2.73 [6-18] | 0.22^a^ |
| **Is your children followed by a dentist?** | |  | |  |  |  |
| No | | 0 (0.0) | | 53 (31.55) | 36 (10.71) | <0.05^b^ |
| Yes, he is followed in a private facility | | 0 (0.0) | | 72 (42.86) | 275 (81.85) |  |
| Yes, he is followed in a public facility | | 0 (0.0) | | 43 (25.60) | 25 (7.44) |  |

*Differences among groups were evaluated with ^a^=Kruskal-Wallis test; ^b^=Chi squared test; ^c^=Fisher's exact test*
